# Porcine iPSC-Derived Intestinal Cells as a High-Fidelity Platform for PEDV Pathogenesis

**DOI:** 10.3390/ani16172718

**Published:** 2026-09-01

**Authors:** Tharathip Muangthong, Warunya Chakritbudsabong, Jarupa Taowan, Phakhin Jantahiran, Wanatchaporn Jaroensuk, Kampon Kaeoket, Sasitorn Rungarunlert

**Affiliations:** 1 Department of Pathobiology, Faculty of Science, Mahidol University, Bangkok 10400, Thailand; tharathip.mua@mahidol.ac.th; 2 Pathobiology Information and Learning Center, Department of Pathobiology, Faculty of Science, Mahidol University, Bangkok 10400, Thailand; 3 Department of Preclinic and Applied Animal Science, Faculty of Veterinary Science, Mahidol University, Nakhon Pathom 73170, Thailand; warunya.cha@mahidol.ac.th (W.C.); phakhin_bin@hotmail.com (P.J.); wanatchaphorncha@gmail.com (W.J.); 4 Laboratory of Cellular Biomedicine and Veterinary Medicine, Faculty of Veterinary Science, Mahidol University, Nakhon Pathom 73170, Thailand; 5 The Monitoring and Surveillance Center for Zoonotic Diseases in Wildlife and Exotic Animals, Faculty of Veterinary Science, Mahidol University, Nakhon Pathom 73170, Thailand; jarupha.tao@mahidol.ac.th; 6 Department of Clinical Sciences and Public Health, Faculty of Veterinary Science, Mahidol University, Nakhon Pathom 73170, Thailand; kampon.kae@mahidol.ac.th

**Keywords:** porcine, intestinal cells, induced pluripotent stem cells, PEDV, viral infection, pathogenesis, infectious model, intestinal model

## Abstract

Porcine epidemic diarrhea virus (PEDV) continues to cause devastating economic losses in the swine industry. However, the lack of species-specific in vitro models and reliance on costly animal models hinders development of effective interventions. We established a high-fidelity platform using porcine induced pluripotent stem cells (piPSCs; line VSMUi001-C) via a 28-day stepwise differentiation protocol. Successful lineage commitment was validated by OCT4 downregulation and robust FOXA2/SOX17 expression at day 5. By day 28, the piPSC-derived intestinal epithelial cells (piPSC-IECs) exhibited organized LGR5+ niches, MUC2+ goblet cells, and Villin+ enterocytes, mirroring native tissue architecture. Barrier functionality was confirmed by the continuous distribution of the tight junction protein zonula occludens-1 (ZO-1). Our results demonstrate that piPSC-IECs are permissive to PEDV and support productive infection, encompassing the complete viral life cycle from entry to release of infectious progeny. Productive replication was confirmed by direct immunodetection of viral antigen within inoculated cells. Viral challenge induced characteristic cytopathic effects and a specific pro-inflammatory elevation of tumor necrosis factor-alpha (TNF-α). Notably, infection fragmented ZO-1, providing a mechanistic explanation for the leaky gut phenotype observed in vivo. This platform serves as a robust system for investigating viral kinetics and host–pathogen interactions with high species-specific relevance.

## Simple Summary

Porcine epidemic diarrhea virus causes significant economic damage to the global swine industry. However, traditional research often relies on non-porcine cell lines or costly animal trials, which fail to accurately mimic natural infection processes. To address this limitation, we developed a high-fidelity intestinal model derived from an in-house porcine induced pluripotent stem cell line. Over the differentiation period, these cells formed a complex tissue containing intestinal stem cells, goblet cells, and mature enterocytes with a functional barrier. This model is permissive to this virus, effectively replicating the viral life cycle and its pathogenesis. Infection induced an immune response and structural fragmentation of the tight junctions. This platform offers a scalable, ethically sustainable tool for screening antivirals and gut supplements, bridging the gap between laboratory research and veterinary applications to improve animal health.

## 1. Introduction

The global swine industry remains under a perpetual state of biosecurity alert due to the evolution and persistence of enteric coronaviruses [1]. Among these, porcine epidemic diarrhea virus (PEDV) represents one of the most significant economic and animal welfare threats. Characterized by severe malabsorptive diarrhea, vomiting, and dehydration, PEDV targets the villous epithelial cells of the neonatal small intestine, often resulting in a high mortality rate in suckling piglets [2,3]. Despite years of research and the deployment of various vaccine strategies, the continuous emergence of highly virulent strains underscores a critical failure to fully elucidate the viral–host interactome within a physiologically relevant context [4,5].

The fundamental bottleneck in PEDV research has been the reliance on suboptimal in vitro models. For decades, the field has leaned heavily on Vero cells (African green monkey kidney), IPEC-J2 (immortalized porcine intestinal) lines and porcine primary enteroids [5,6,7]. These models permit viral replication and demonstrate the lethal pathogenesis. However, they contain inherent limitations stemming from significant biological inaccuracies, especially the lack of host–pathogen specificity, the absence of the heterogeneous and complex microenvironment of the porcine gut, and a limited proliferative capacity [8]. Moreover, in vivo studies are limited by ethical burdens [9].

The advent of porcine induced pluripotent stem cell (piPSC) technology offers a transformative solution. By reprogramming somatic cells into a pluripotent state, we can generate a theoretically infinite source of porcine-specific genetic material that retains the capacity for multilineage differentiation [10]. Porcine iPSCs enable the construction of intestinal tissues that mimic in vivo architecture more accurately than traditional models [9,11]. However, establishing viral infection remains a significant hurdle due to potential toxicity and gut barrier dysfunction. To overcome these limitations, a validated, high-fidelity viral pathogenesis model is necessary.

In this study, we report the successful derivation of piPSC-derived intestinal epithelial cells (piPSC-IECs) from our in-house piPSC line (VSMUi001-C) and evaluate their utility as a high-fidelity platform for investigating PEDV pathogenesis and host immune responses. By bridging the gap between traditional cell lines and costly animal models, this piPSC-based platform provides a scalable, ethically sustainable, and physiologically relevant tool for characterizing viral–host interactions and screening next-generation antiviral therapeutics.

## 2. Materials and Methods

### 2.1. Ethics Statement

The study followed strict ethical guidelines for animal procurement and use. It was approved by the Animal Ethics Committee at the Faculty of Veterinary Science, Mahidol University in Thailand, under ethics approval number MUVS 2022-03-10. The reporting of animal experiments adheres to the ARRIVE (Animal Research: Reporting of In Vivo Experiments) guidelines 2.0.

### 2.2. Animals

Pregnant ICR mice, approximately 13.5 days postcoitum, were obtained from Nomura Siam International Co., Ltd., Bangkok, Thailand. The isolation of mouse embryonic fibroblasts, used as feeder cells, adhered to methodologies outlined in earlier studies [12].

### 2.3. Porcine iPSC Culture

The previously characterized piPSC line, VSMUi001-C, was utilized for all experiments [10]. The maintenance and culture of this piPSC line followed modified protocols adapted from previous publications. Briefly, the piPSCs were maintained on a feeder layer of mitomycin C-inactivated mouse embryonic fibroblasts. The culture medium consisted of DMEM/F-12 (cat. no. 12500062, Gibco, Waltham, MA, USA) supplemented with 20% Knockout™ Serum Replacement (cat. no. 10828-028, Gibco), 0.1 mM 2-mercaptoethanol (cat. no. 21985-023, Gibco), 1% antibiotic–antimycotic solution (cat. no. 15240062, Gibco), 1% GlutaMAX™ (cat. no. 35050061, Gibco), 1% MEM non-essential amino acids (cat. no. 11140-050, Gibco), 2 µM SB431542 (cat. no. 1614/1, Tocris, Bristol, UK), 3 µM CHIR99021 (cat. no. 4423/10, Tocris), and 1000 U/mL mouse leukemia inhibitory factor (cat. no. ESG1107, Millipore, Burlington, MA, USA). The piPSCs were passaged every two days by detaching colonies using TrypLE™ Select (cat. no. A1217701, Gibco) at 37 °C for 1 min, with daily medium replacement. Cells between passages 20 and 35 were used for all subsequent differentiation experiments.

### 2.4. Differentiation of piPSC-IECs

The differentiation protocol for piPSCs was adapted from previous studies [13,14]. The piPSCs were seeded at a density of 1.35 × 10^5^ cells/well into 6-well plates containing loose feeder cells (approximately 1:1 ratio) and incubated for 2 days with daily medium replacement. Induction of definitive endoderm (DE) was performed using a basal culture medium consisting of DMEM/F-12 (cat. no. 12500062, Gibco), supplemented with 15% fetal bovine serum (cat. no. SV30160.03, HyClone, Pasching, Austria), 0.1 mM 2-mercaptoethanol (cat. no. 21985-023, Gibco), 1% antibiotic–antimycotic (cat. no. 15240062, Gibco), 1% GlutaMAX™ (cat. no. 35050061, Gibco), and 1% MEM non-essential amino acids (cat. no. 11140-050, Gibco). This medium was further supplemented with 25 ng/mL Activin A (cat. no. 338-AC, R&D Systems, Minneapolis, MN, USA) or Medium I for 5 days.

Subsequently, differentiation into immature intestinal cells was continued by treating the cells with basal medium supplemented with 5 µM 6-bromoindirubin-3′-oxime (6-BIO) (cat. no. 3194/10, Tocris) and 10 µM of the Notch inhibitor DAPT (cat. no. 2634/10, Tocris) (Medium II). Following this 9-day induction period, the culture was transitioned to Medium III, which consisted of the basal medium enriched with 100 ng/mL h-EGF (cat. no. 236-EG, R&D Systems), 100 ng/mL Noggin (cat. no. 6057-NG, R&D Systems), and 500 ng/mL R-spondin-1 (cat. no. 4645-RS, R&D Systems). Cells were maintained in Medium III for an additional 14 days (from day 15 to day 28) with half-medium changes every two days. The differentiation stage was verified via immunofluorescence staining for lineage-specific markers. The resulting mature piPSC-IECs were expanded and cryopreserved at −80 °C (or in liquid nitrogen) until further use.

### 2.5. PEDV Preparation and Propagation

The PEDV isolate was provided by the Monitoring and Surveillance Center for Zoonotic Diseases in Wildlife and Exotic Animals (MoZWE), Faculty of Veterinary Science, Mahidol University, Thailand. For virus preparation, Vero cells (ATCC CCL-81, Manassas, VA, USA) were maintained in EMEM supplemented with 10% heat-inactivated FBS and antibiotics and incubated at 37 °C with 5% CO_2_. Upon reaching appropriate confluency, cells were sub-cultured using 0.125% trypsin-EDTA to prepare monolayers for infection.

For virus isolation, cell monolayers were washed twice with TPCK-MEM and inoculated with samples, followed by adsorption for 2 h at 37 °C. The inoculum was subsequently removed and replaced with maintenance medium (EMEM supplemented with 2 µg/mL TPCK, 0.3% TPB, and 0.02% yeast extract), and cells were incubated for 72 h. Cytopathic effects (CPEs) were monitored every 24 h; if no CPE was observed, blind passage was performed for up to five passages. Viral presence was confirmed via PCR. The primer pair specific to the PEDV gene sequence consisted of the forward primer PEDV1265F (5′-CCA AAG ACG ACC TCG CTT GC-3′) and the reverse primer PEDV2035R (5′-CGT CAG CAT CAG CAA GAT GTC TG-3′) (Figure S1).

For virus propagation, confluent Vero cells were washed twice with TPCK-MEM followed by inoculation of the virus onto the cells. The cultures were incubated at 37 °C in a 5% CO_2_ atmosphere for 2 h. The inoculum was replaced with maintenance medium containing 2% FBS, and cells were incubated for 72 h with daily observation for CPEs. Upon the development of clear CPEs, infection was re-confirmed by PCR. Viral supernatants were then collected, stored at −80 °C, and titrated using a plaque assay. The specific PEDV at passage 16, with a stock titer of 1.1 × 10^6^ plaque-forming units per milliliter (PFU/mL), was utilized for all subsequent downstream investigations.

### 2.6. Plaque Assay

Vero cells were seeded in 6-well plates and incubated at 37 °C with 5% CO_2_ until reaching 90–100% confluency. Viral samples were serially ten-fold diluted in serum-free medium. The cell monolayers were washed once with PBS, and 200 µL of each virus dilution were inoculated onto the cells. Plates were gently rocked to ensure even distribution and incubated for 1–2 h at 37 °C to allow viral adsorption. After incubation, the inoculum was removed, and cells were overlaid with maintenance medium containing 1–2% FBS and 1–1.5% agarose or carboxymethyl cellulose to restrict viral spread. Plates were then incubated at 37 °C with 5% CO_2_ for 2–4 days until visible plaques formed.

Following incubation, cells were fixed with 4% paraformaldehyde and stained with 0.1% crystal violet to visualize plaques. The overlay was removed, and plaques were counted in wells containing 10–100 plaques. Viral titers were calculated and expressed as PFU/mL using the formula: PFU/mL = (number of plaques × dilution factor)/volume of inoculum (mL) [15].

### 2.7. PEDV Infection of piPSC-IECs

The piPSC-IECs were thawed and cultured with the basal culture medium (DMEM/F-12 (12500062, Gibco, Waltham, MA, USA), enhanced with 15% FBS (cat. no. SV30160.03, HyClone), 0.1 mM 2-mercaptoethanol (21985-023, Gibco), 1% antibiotic–antimycotic solution (15240062, Gibco), 1% GlutaMAX™ (35050061, Gibco), 1% MEM non-essential amino acids (11140-050, Gibco)). The cells were subcultured for the experiments using 0.25% trypsin and seeded into a 6-well plate at a density of 1 × 10^6^ cells/well and incubated for 24 h at 37 °C in a humidified atmosphere containing 5% CO_2_. PEDV, at a multiplicity of infection (MOI) of 0.1, was inoculated onto the piPSC-IECs for 2 h, then washed three times with PBS and refreshed with basal medium. CPEs were monitored daily. Upon the onset of observable CPEs (48 h post-inoculation, hpi), culture supernatants and cell lysates were harvested for viral titration via plaque assay.

### 2.8. Immunofluorescence Staining and Imaging Analysis

To identify specific markers for intestinal cell lineages and cytokine response, the piPSC-IECs were cultured on cover glass with and without co-culture with PEDV. The samples were initially fixed in 4% paraformaldehyde for 15 min. Following fixation, they were permeabilized with 0.25% Triton-X 100 in PBS at 37 °C for 10 min. To reduce non-specific binding, the piPSC-IECs were incubated in a 2% bovine serum albumin solution in PBS at 4 °C for 1 h. Primary antibodies were then applied and incubated overnight at 4 °C in the dark (Table 1). This was followed by a 1 h incubation at 37 °C with secondary antibodies conjugated with DAPI. After staining, the piPSC-IECs were mounted on microscope slides using an antifade mounting medium. Visualization was conducted using a Leica DMi8 inverted fluorescence microscope (Leica Microsystems, Wetzlar, Germany) equipped with a Leica DFC7000 camera (Leica Microsystems). For each sample, a minimum of 40 z-stacks were acquired at intervals of 0.1 µm. All images were processed using the Leica Application Suite X (LAS X) imaging software (version 3.7.6.25997; Leica Microsystems) and analyzed for fluorescence intensity with ImageJ software (version 1.54g, National Institutes of Health, Bethesda, MD, USA; https://imagej.nih.gov/ij/). For each condition, six randomly selected, non-overlapping fields were analyzed, and the mean fluorescence intensity per field was measured using identical acquisition, exposure and thresholding settings across all conditions.

### 2.9. Statistical Analysis

Differentiation and infection experiments were performed in three independent biological replicates. For image-based quantification, six randomly selected fields per condition were analyzed (*n* = 6), and the mean fluorescence intensity per field was measured in ImageJ. These data are presented as box plots showing the median, interquartile range and full data range, with individual field values overlaid. Differences between mock- and PEDV-inoculated groups were assessed using an unpaired Student’s *t*-test after confirming normality with the Shapiro–Wilk test. All statistical analyses were performed using SPSS version 25.0 (IBM Corp., Armonk, NY, USA). A *p*-value of less than 0.05 was considered statistically significant.

## 3. Results

### 3.1. Differentiation and Characterization of piPSC-IECs

**Figure 1:**
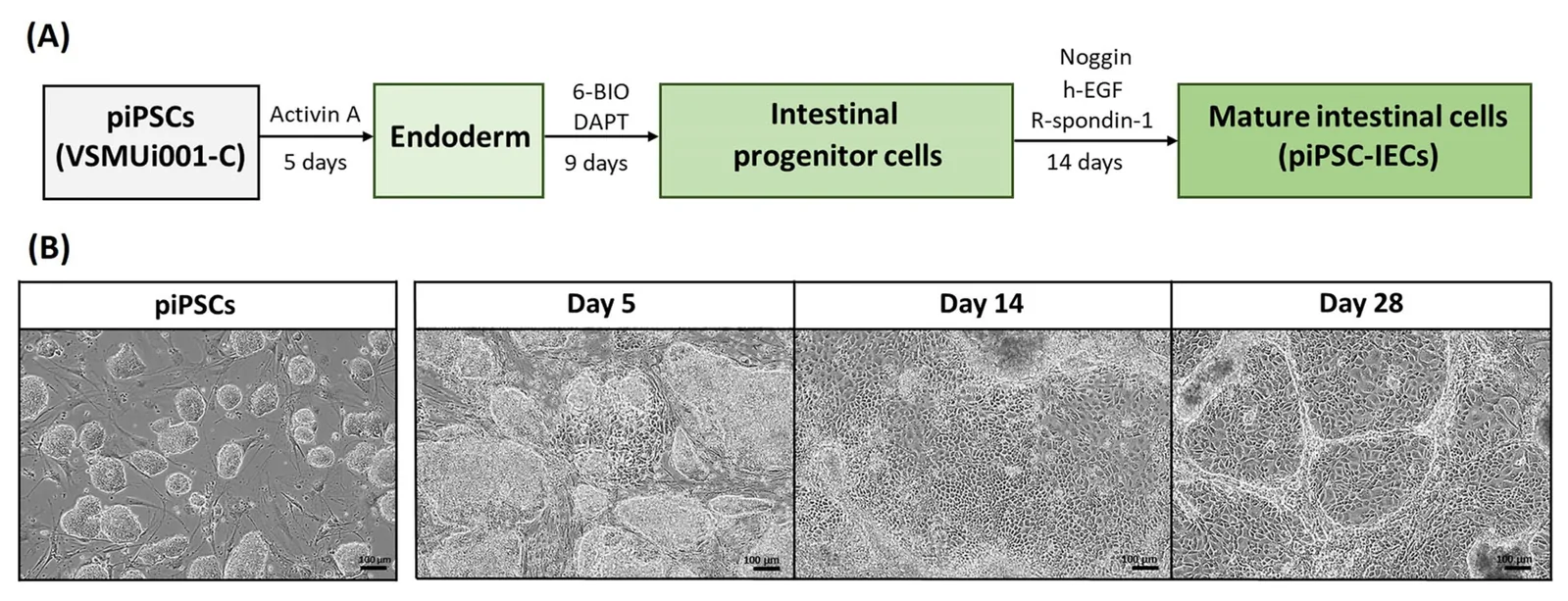
Stepwise differentiation of piPSCs into functional intestinal epithelial cells. (**A**) Schematic representation of the stepwise induction protocol used to direct piPSCs toward the porcine intestinal epithelial cell (piPSC-IEC) lineage. (**B**) Representative phase-contrast images illustrating the morphological evolution of the culture milestones: pluripotent piPSCs exhibiting characteristic dome-like colonies; definitive endoderm (DE) at day 5 showing a transition to a flattened, cobblestone-like appearance; immature progenitor cells (days 9–14); and mature piPSC-IECs at day 28, characterized by the epithelial-like morphology and the development of distinct cord-like structures. Scale bars, 100 µm.

The differentiation of piPSCs into porcine intestinal epithelial cells (piPSC-IECs) was successfully induced through a stepwise protocol (Figure 1A). Initially, piPSCs were directed toward the DE lineage. By day 5 of induction, the cells exhibited a distinct morphological transition from characteristic dome-like colonies to a flattened, cobblestone-like appearance. Following subsequent treatment with lineage-specific media to generate immature cells (days 9–14), the culture was maintained until day 28 to reach full maturity. At this terminal stage, the culture was characterized by the epithelial-like morphology and the development of distinct cord-like structures (Figure 1B).

**Figure 2:**
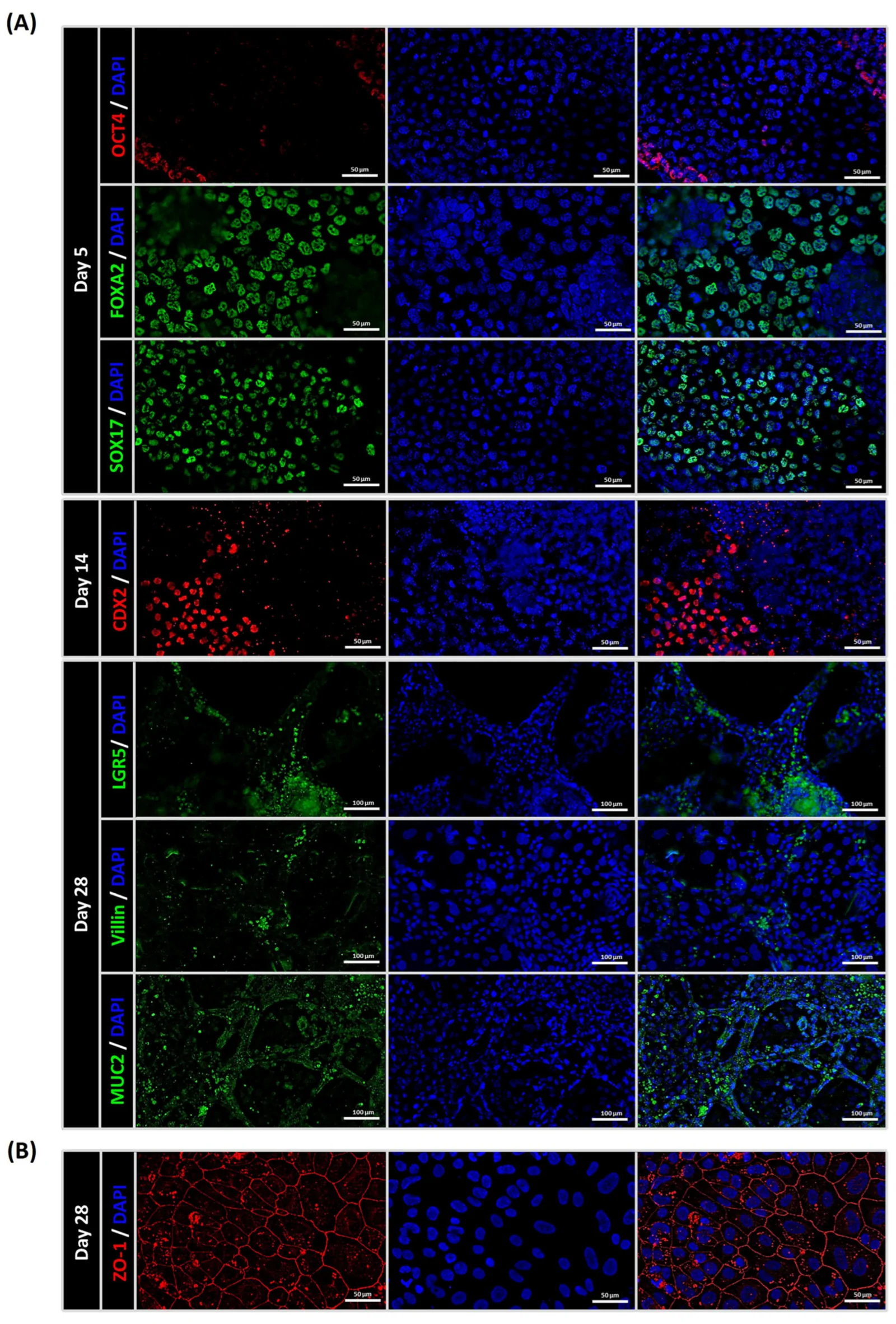
Characterization of piPSC-IEC differentiation milestones via immunofluorescence. (**A**) Immunofluorescence analysis of lineage-specific markers throughout the differentiation protocol. At day 5, cells exhibit robust expression of the definitive endoderm (DE) markers FOXA2 and SOX17, concurrent with reduced expression of the pluripotency marker OCT4, confirming successful lineage commitment. By day 14, cells express the intestinal progenitor marker CDX2. At day 28, the culture displays specialized markers of mature intestinal epithelium, including LGR5 (intestinal stem cells), Villin (mature enterocyte microvilli), and MUC2 (goblet cells). Scale bars, 50 µm (day 5 and day 14) and 100 µm (day 28). (**B**) Representative immunofluorescence images of the tight junction protein (ZO-1) at day 28. The continuous, well-defined distribution of ZO-1 along cell–cell boundaries indicates the establishment of a robust junctional architecture and a functional epithelial barrier. Scale bar, 50 µm.

To verify the successful establishment of the targeted lineages, immunofluorescence (IF) analysis was performed at key milestones. At day 5, the cells exhibited dominant expression of the DE markers FOXA2 and SOX17, while the pluripotency marker OCT4 was significantly reduced, confirming successful lineage commitment. By day 14, the presence of the intestinal progenitor marker CDX2 was detected, validating the transition into an immature intestinal lineage. Finally, at day 28, the expression of specialized intestinal markers was assessed, including LGR5 (intestinal stem cells), MUC2 (goblet cells), and Villin (mature enterocyte microvilli). The resulting differentiated tissue showed robust expression of LGR5, which was predominantly localized within organized cord-like structures. In contrast, MUC2 and Villin exhibited a more generalized, scattered distribution throughout the tissue, confirming the presence of diverse, specialized intestinal cell types (Figure 2A).

To confirm the structural integrity of the intestinal barrier, the expression and localization of the tight junction protein zonula occludens-1 (ZO-1) were investigated. At day 28, the mature cells exhibited a continuous, well-defined distribution of ZO-1 along the cell–cell boundaries, representing the establishment of a robust junctional architecture characteristic of a functional epithelial barrier (Figure 2B).

Collectively, these morphological and phenotypic characteristics demonstrate that the stepwise induction protocol and conditioned media effectively drove the differentiation of piPSCs into functional, high-fidelity porcine intestinal epithelium.

### 3.2. Susceptibility of piPSC-IECs to PEDV Infection and Viral Replication

**Figure 3:**
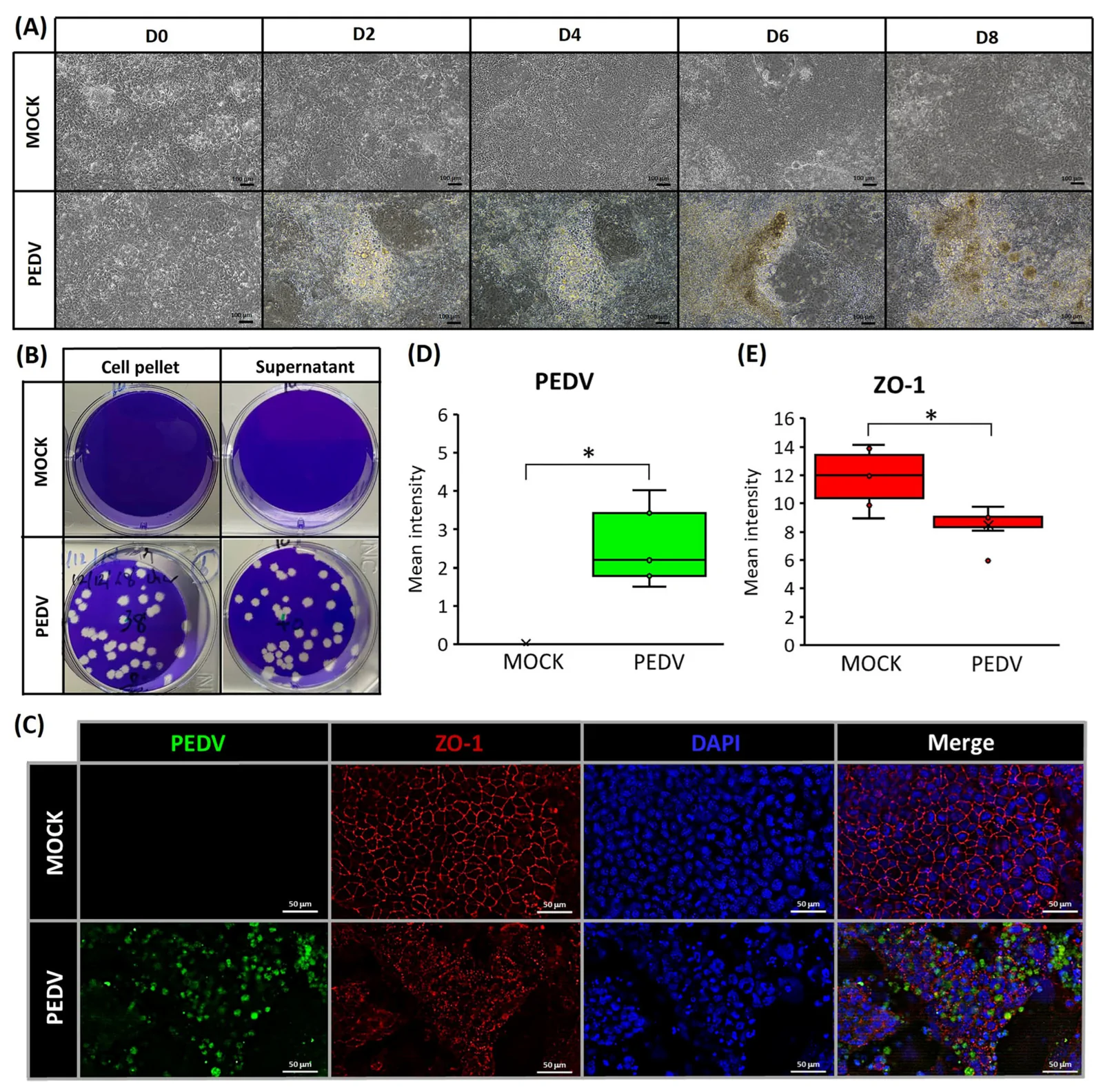
Permissiveness of piPSC-IECs to PEDV infection and subsequent compromise of epithelial barrier integrity. (**A**) Time-course observation of cytopathic effects (CPEs) following PEDV inoculation (MOI = 0.1). Morphological alterations initially manifest as characteristic cell swelling at 2 days post-inoculation (dpi), progressing to extensive cell clumping and widespread cell death by 6–8 dpi. Scale bars, 100 µm. (**B**) Viral titration via plaque assay from harvested cell pellets (intracellular) and culture supernatants (extracellular) harvested at 48 hpi. The detection of infectious viral particles in both fractions confirms active viral replication and the successful release of infectious progeny within the piPSC-IEC model. (**C**) Representative immunofluorescence images of the PEDV (green), tight junction protein zonula occludens-1 (ZO-1, red), and nuclei (DAPI, blue) in mock-infected (MOCK) and PEDV-infected piPSC-IECs at 48 hpi. Scale bars, 50 µm. (**D**,**E**) Quantitative analysis of fluorescence intensity of PEDV and ZO-1 in infected cells compared with MOCK controls. Box plots show the median, interquartile range and full data range, with individual field values overlaid (*n* = 6 randomly selected fields per condition). Statistical significance between groups was determined using an unpaired Student’s *t*-test. Asterisks (*) indicate statistically significant differences between groups (*p* < 0.05).

To evaluate the suitability of the piPSC-IECs as a high-fidelity platform for viral studies, the differentiated cells were challenged with PEDV to assess their susceptibility to infection. CPEs were initially observed at 2 days post-inoculation (dpi), primarily manifesting as characteristic cell swelling. The infection progressed significantly over time; by 6–8 dpi, extensive cell clumping and widespread cell death were recorded (Figure 3A).

To verify that the observed CPE was a result of active viral replication and dissemination, both the cell pellets and the culture supernatants were harvested for titration via plaque assay. The presence of infectious viral particles was confirmed in both the intracellular (cell pellet) and extracellular (supernatant) fractions, demonstrating that piPSC-IECs support the full viral life cycle from initial entry to the assembly and release of infectious progeny (Figure 3B, Table 2). Direct immunodetection of PEDV antigen within these cells, confirming cellular susceptibility to viral infection rather than residual input virus, is presented in Section 3.3 (Figure 3C). Collectively, these findings indicate that the piPSC-IEC model effectively recapitulates key features of PEDV pathogenesis and provides a robust, porcine-specific system for investigating infectious viral progeny production.

### 3.3. Confirmation of PEDV Protein Expression and Its Impact on Intestinal Barrier Integrity

To evaluate the impact of PEDV on the integrity of the intestinal barrier, the expression and localization of the tight junction protein ZO-1 were investigated. Under control conditions, mock-infected piPSC-IECs exhibited a continuous, well-defined distribution of ZO-1 along the cell–cell boundaries, with no detectable PEDV signal. Following inoculation, piPSC-IECs displayed a robust, punctate cytoplasmic PEDV immunofluorescence signal. Because the inoculum was removed and the epithelial constructs were washed three times with PBS before the observation period, any residual input virions were eliminated; the antigen detected therefore represents viral protein synthesized de novo within the piPSC-IECs. Together with the recovery of infectious progeny from both the cell and supernatant fractions (Table 2), this confirms that the piPSC-IEC platform supports a complete and productive PEDV replication cycle. Notably, areas of high viral accumulation directly correlated with widespread structural fragmentation of the ZO-1 junctional architecture. Furthermore, quantitative analysis of fluorescence in PEDV-inoculated cells revealed a strong viral signal accompanied by a significant reduction in ZO-1 intensity (Figure 3C–E). Collectively, these results demonstrate that PEDV infection directly compromises the epithelial barrier by disrupting key junctional complexes, further validating the piPSC-IEC platform as a high-fidelity model for studying the cellular pathogenesis of porcine enteric viruses.

### 3.4. Pro-Inflammatory Cytokine Response of piPSC-IECs to PEDV Infection

**Figure 4:**
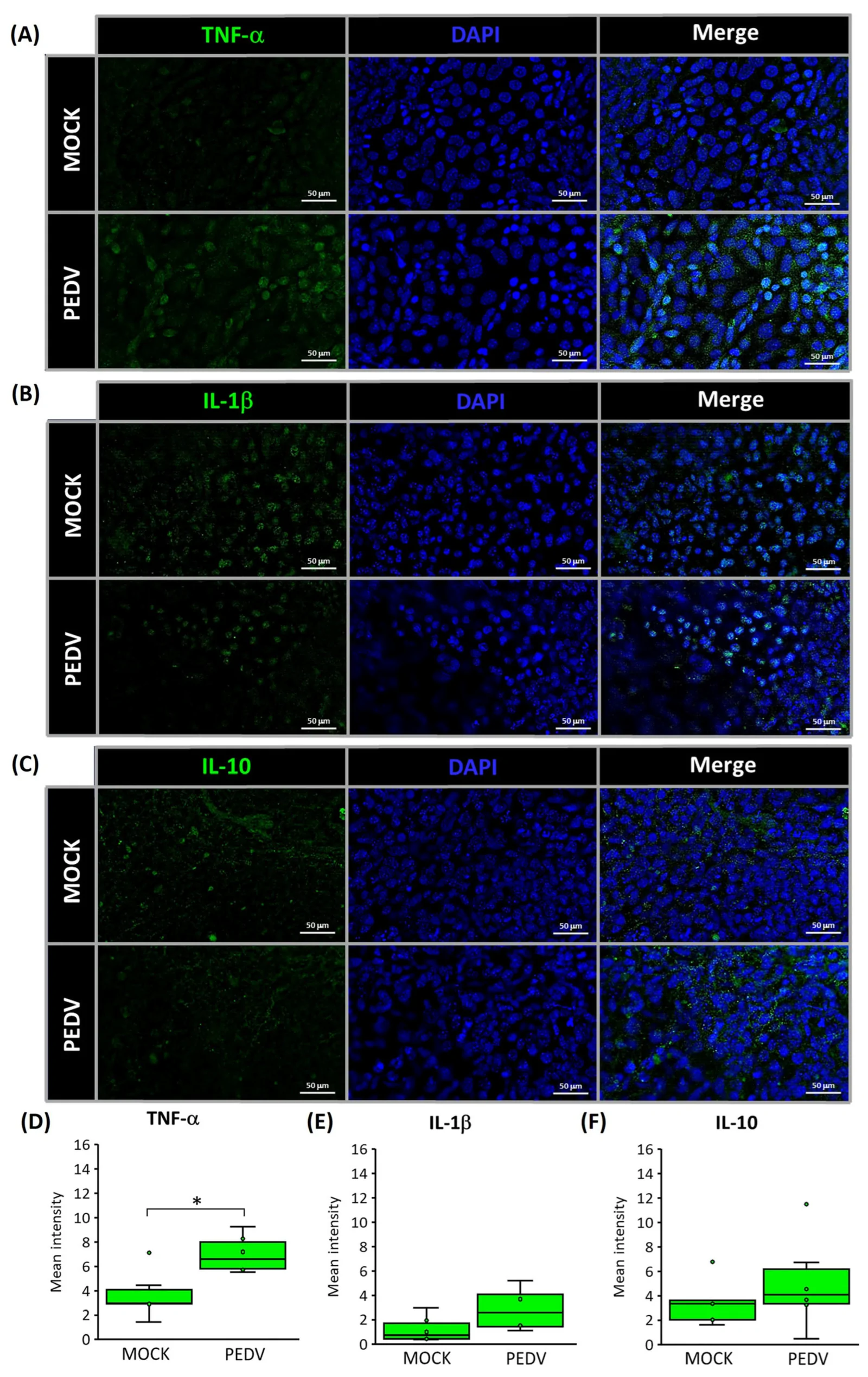
Innate immune signaling in piPSC-IECs during viral challenge. (**A**–**C**) Immunofluorescence detection of key cytokines, including tumor necrosis factor-alpha (TNF-α), interleukin-1 beta (IL-1β), and interleukin-10 (IL-10), following PEDV inoculation at 48 hpi. Scale bars, 50 µm. (**D**–**F**) Quantitative analysis of fluorescence intensity showed significantly increased TNF-α expression in infected cells compared with MOCK controls (*p* < 0.05), while IL-1β and IL-10 expression levels were not significantly different. Box plots show the median, interquartile range and full data range, with individual field values overlaid (*n* = 6 randomly selected fields per condition). Statistical significance between groups was determined using an unpaired Student’s *t*-test. Asterisks (*) indicate statistically significant differences between groups (*p* < 0.05).

To evaluate the innate immune response of the porcine intestinal epithelial platform during viral challenge, the expression of key cytokines was assessed via IF microscopy. Following inoculation with PEDV, piPSC-IECs were examined for the immunoreactivity of tumor necrosis factor-alpha (TNF-α), interleukin-1 beta (IL-1β), and interleukin-10 (IL-10) (Figure 4A–C).

Quantitative analysis of the fluorescence intensity revealed that TNF-α expression was significantly elevated in PEDV-infected cells compared to the mock-infected (MOCK) control group (*p* < 0.05). In contrast, no significant differences were observed in the expression levels of IL-1β or IL-10 between the infected and mock conditions (Figure 4D–F). These findings indicate that piPSC-IECs are capable of mounting a specific pro-inflammatory response characterized by TNF-α expression, further validating the ability of this model to recapitulate the immunological features of PEDV pathogenesis in the porcine intestine.

## 4. Discussion

The establishment of a high-fidelity, porcine-specific model is critical for overcoming the limitations of traditional cell cultures and costly animal models in studying enteric pathogens. PEDV primarily targets the intestinal villous epithelial cells in vivo through fecal-oral transmission [1,16]. While established models such as Vero, MARC-145, and HEK293 cells facilitate viral propagation, they lack species-specific physiological relevance [7,17]. Furthermore, although the nontransformed porcine line IPEC-J2 is available, it fails to recapitulate the complex spatial architecture and diverse cellular composition of the natural intestinal epithelium [18]. This study demonstrates the successful generation of piPSC-IECs from the VSMUi001-C line, which not only recapitulates the multicellular complexity of the porcine intestine but also serves as a permissive platform for investigating PEDV pathogenesis and innate immune signaling.

A key strength of this platform is the 28-day stepwise differentiation protocol, which directs piPSCs through a DE stage toward a mature intestinal phenotype. The transition was validated by the significant downregulation of the pluripotency marker, OCT4, alongside the robust expression of FOXA2 and SOX17, confirming successful lineage commitment [19,20]. Unlike rapid differentiation protocols, our extended maturation period allowed for the development of distinct cord-like structures and the expression of specialized markers, including LGR5+ stem cells, MUC2+ goblet cells, and Villin+ enterocytes. The localized expression of LGR5 within these cords suggests the formation of primitive crypt-like niches, providing a more physiologically relevant environment than standard immortalized lines [18,21]. Furthermore, the presence of MUC2+ goblet cells and Villin+ enterocytes confirm the development of mature intestinal epithelial cells capable of mucus secretion and Actin dynamic regulation, both essential for maintaining the mucosal barrier against enteric pathogens [22,23].

Our results confirm that piPSC-IECs are permissive to PEDV and support productive infection, encompassing the complete viral life cycle from initial entry to the release of infectious progeny. In vivo studies indicate that PEDV primarily targets mature enterocytes, causing epithelial damage and villous atrophy that led to severe diarrhea [24]. Our platform demonstrates a correlation between enterocyte marker expression (Villin+) and the manifestation of CPEs, specifically cell swelling, clumping, and detachment, which mirror those observed in the porcine gut in vivo [25,26]. Moreover, PEDV demonstrated efficient replication kinetics in the newly established piPSC-IECs, reaching titers of 3.65 × 10^3^ PFU/mL (supernatant) and 3.55 × 10^3^ PFU/mL (pellet) at 48 hpi under trypsin-free conditions (Table 2). Although this propagation magnitude is lower than the peak titer observed in standard Vero cells (1.1 × 10^6^ PFU/mL at 72 hpi), the trypsin-independent susceptibility of piPSC-IECs highlights its physiological accuracy as a porcine intestinal model compared to the artificial expansion seen in non-porcine cell lines. Notably, PEDV infection directly compromised the integrity of the intestinal barrier, as evidenced by the structural fragmentation of the tight junction protein ZO-1 in infected cells related to the piglet-infected condition [27]. Previous research utilizing IPEC-J2 cells reported that PEDV utilizes the junctional protein Occludin as an entry factor, leading to its internalization and subsequent barrier failure, though Claudin-1 remained largely unaffected [16]. Our observation of ZO-1 disruption provides a complementary mechanistic explanation for the leaky gut phenotype and severe dehydration that define PEDV clinical pathogenesis, validating our platform as a high-fidelity model for investigating genuine PEDV infection dynamics.

According to previous studies, PEDV infection induces inflammatory responses in IPEC-J2 cells, although the magnitude and profile of cytokine induction may vary depending on the viral strain and experimental conditions [4,28,29]. In the present study, PEDV-infected piPSC-IECs exhibited significant upregulation of TNF-α, whereas no significant changes were observed in IL-1β or IL-10 expression. While direct comparisons should be interpreted with caution due to differences in experimental design, these findings suggest that piPSC-IECs are capable of mounting a measurable pro-inflammatory response to PEDV infection. The robust elevation of TNF-α aligns with previous reports of PEDV-induced acute pro-inflammatory signaling in intestinal epithelium [30]. While intracellular levels of IL-1β and IL-10 remained largely unchanged during early infection, quantifying cytokine secretion profiles in the culture supernatant remains an essential direction for future studies to comprehensively characterize the host immune response [31,32]. Collectively, these observations support the utility of the piPSC-IEC model for investigating host–pathogen interactions and epithelial responses to enteric viral infection. Future studies directly comparing piPSC-IECs and IPEC-J2 cells under identical infection conditions, including viral replication kinetics and cytokine profiling, will be valuable for defining the relative strengths and physiological relevance of each model.

While the piPSC-IEC model offers significant advantages, we acknowledge certain limitations. First, the cells were primarily maintained in a 2D+ format; while cord-like structures formed, they may not fully capture the 3D complexity of whole organoids embedded in an extracellular matrix [9,21,33]. Second, the use of −80 °C storage for mature cells, while convenient for screening, requires further optimization compared to liquid nitrogen to ensure long-term viability [34]. Another limitation of this study is that only a single PEDV isolate was evaluated, and genotypic characterization of the virus was not performed. Consequently, the broader applicability of the findings across genetically diverse PEDV strains remains to be determined. However, the primary objective of this study was to establish and provide proof-of-concept validation of the piPSC-IEC platform as an in vitro model for PEDV infection. Therefore, we focused on a field isolate obtained from a regional surveillance program to evaluate the susceptibility of piPSC-IECs to productive PEDV infection. Future studies incorporating S-gene sequencing, phylogenetic analysis, and the evaluation of genetically diverse PEDV isolates will be important for further characterizing the viral strain used in this study, determining the broader applicability of the piPSC-IEC platform, and assessing potential genotype-dependent differences in viral replication, cellular tropism, and host responses. To further resolve the functional characteristics of this model, future work will integrate functional permeability assays, such as transepithelial electrical resistance (TEER), to precisely quantify epithelial barrier dynamics during infection [35].

Although the term ‘high-fidelity’ must be interpreted within the context of epithelial biology, the piPSC-IEC model successfully reproduces key features of PEDV infection observed in vivo, including epithelial cell heterogeneity, barrier disruption, CPEs, viral localization and inflammatory responses. Nonetheless, the current platform does not fully replicate the complexity of the native intestinal microenvironment, such as crypt-villus architecture, immune cell interactions, vascularization, and dynamic mucus and fluid flow. Future efforts will prioritize the integration of Transwell-based systems, organoid-derived architectures, and immune co-culture approaches to enhance the physiological relevance of this model. Collectively, these observations suggest that the piPSC-IEC model provides a promising platform for investigating PEDV–host interactions; however, direct comparisons with established intestinal epithelial models under identical experimental conditions are warranted to further validate these advantages.

## 5. Conclusions

In conclusion, this study reports the successful establishment of a high-fidelity, porcine-specific intestinal platform derived from the VSMUi001-C piPSC line. Through a robust 28-day stepwise differentiation protocol, we generated a multicellular intestinal epithelium that recapitulates the complex architecture of the native porcine gut, including the presence of LGR5 stem cell niches, MUC2+ goblet cells, and Villin+ enterocytes. The establishment of a functional epithelial barrier was validated by the continuous and well-defined distribution of the tight junction protein ZO-1.

Our findings demonstrate that this piPSC-IEC model is permissive to PEDV and supports productive infection, encompassing the complete viral life cycle and effectively mirroring the in vivo clinical pathogenesis. The observed structural fragmentation of ZO-1 and the specific pro-inflammatory elevation of TNF-α provide a crucial mechanistic link between viral replication and the leaky gut phenotype observed in infected swine. By bridging the gap between traditional cell lines and costly animal trials, this piPSC-based platform provides a scalable, ethically sustainable, and physiologically relevant tool for the screening of next-generation antivirals and probiotics to enhance intestinal resilience in the swine industry.

## Figures and Tables

**Table 1:** Primary and secondary antibodies.

Antibodies	Dilution	Companies	Cat #
Goat anti-OCT4	1:200	Santa Cruz Biotechnology, Dallas, TX, USA	SC-8628
Goat anti-SOX17	1:200	R&D Systems	AF1924
Goat anti-FOXA2	1:200	R&D Systems	AF2400
Rabbit anti-CDX2	1:500	Abcam, Cambridge, UK	Ab88129
Mouse anti-VILLIN	1:500	Santa Cruz Biotechnology	SC-58897
Rabbit anti-MUCIN2	1:100	Biorbyt, Cambridge, UK	orb372331
Rabbit anti-LGR5	1:100	Novus Biologicals, Centennial, CO, USA	NBP1-28904
Rabbit anti-ZO-1	1:100	Thermo Fisher Scientific, Waltham, MA, USA	61-7300
Mouse anti-PEDV	1:500	Abcam	Ab325989
Goat anti-porcine TNF-α	1:100	R&D Systems	AF690
Mouse anti-porcine IL-1β	1:100	R&D Systems	MAB6811
Goat anti-porcine IL-10	1:100	R&D Systems	BAF-693
Alexa Fluor 488 donkey anti-goat IgG	1:2000	Thermo Fisher Scientific	A-11055
Alexa Fluor 488 donkey anti-mouse IgG	1:2000	Thermo Fisher Scientific	A32766TR
Alexa Fluor 488 donkey anti-rabbit IgG	1:2000	Thermo Fisher Scientific	A-21206
Alexa Fluor 594 donkey anti-goat IgG	1:2000	Thermo Fisher Scientific	A-11058
Alexa Fluor 594 donkey anti-rabbit IgG	1:2000	Thermo Fisher Scientific	A-21207

**Table 2:** PEDV titers of intracellular (cell pellet) and extracellular (supernatant) fractions in the piPSC-IEC model.

piPSC-IEC	PEDV Titer (PFU/mL)	
	Cell Pellet	Supernatant
**Mock**	0.00	0.00
**PEDV**	3.55 × 10^3^	3.65 × 10^3^

## Data Availability

The original contributions presented in this study are included in the article/Supplementary Material. Further inquiries can be directed to the corresponding author.

## Supplementary Materials

The following supporting information can be downloaded at: https://www.mdpi.com/article/10.3390/ani16172718/s1, Figure S1. Detection of PEDV by PCR. A representative agarose gel electrophoresis image showing amplification of the PEDV-specific fragment using the forward primer PEDV1265F (5′-CCA AAG ACG ACC TCG CTT GC-3′) and the reverse primer PEDV2035R (5′-CGT CAG CAT CAG CAA GAT GTC TG-3′). The expected amplicon size was approximately 770 bp, confirming the presence of PEDV RNA in the sample.

## Abbreviations

The following abbreviations are used in this manuscript:

CPE	cytopathic effect
DAPI	4′,6-diamidino-2-phenylindole
DE	definitive endoderm
dpi	days post-inoculation
hpi	hours post-inoculation
IF	immunofluorescence
IL-1β	interleukin-1 beta
IL-10	interleukin-10
MOI	multiplicity of infection
PEDV	Porcine epidemic diarrhea virus
PFU	plaque-forming units
piPSCs	porcine induced pluripotent stem cells
piPSC-IECs	piPSC-derived intestinal epithelial cells
TEER	transepithelial electrical resistance
TNF-α	tumor necrosis factor-alpha
ZO-1	zonula occludens-1
