# Supplementary material for: Porcine iPSC-Derived Intestinal Cells as a High-Fidelity Platform for PEDV Pathogenesis
Source: Animals (Basel). 2026 Sep 1;16(17):2718. doi: 10.3390/ani16172718 (PMC13565315; doi:10.3390/ani16172718)
Supplement: Supplementary file 1 [file animals-16-02718-s001.zip › animals-4348548-supplementary.pdf]

## Supplementary Figure S1.

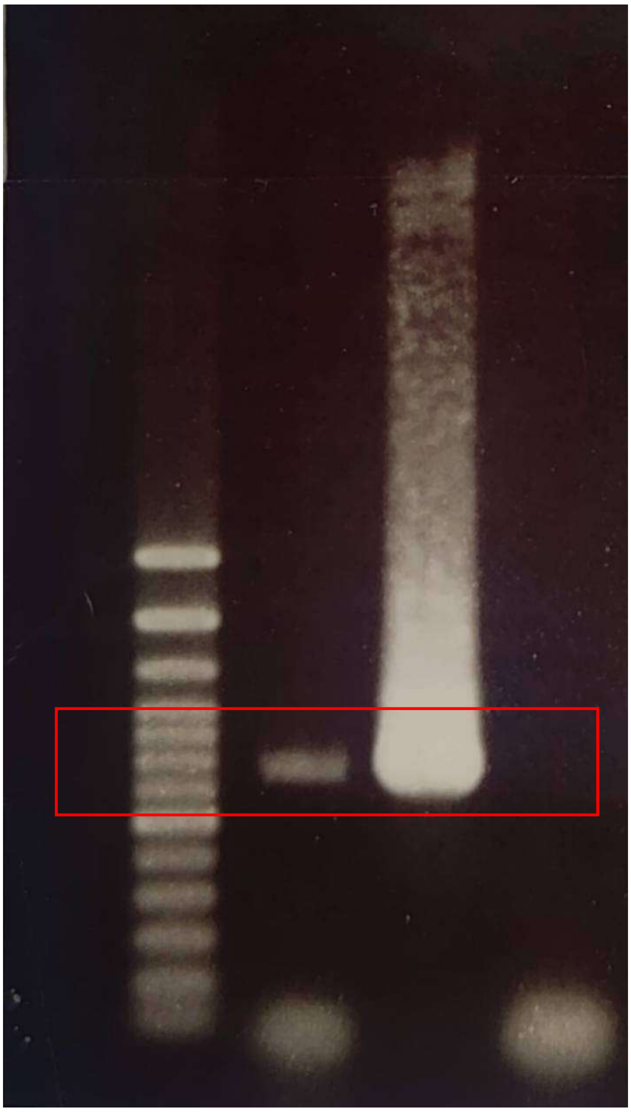


**Supplementary Figure S1. Detection of PEDV by PCR.**
A representative agarose gel electrophoresis image showing amplification of the PEDV-specific fragment using the forward primer PEDV1265F (5′-CCA AAG ACG ACC TCG CTT GC-3′) and the reverse primer PEDV2035R (5′-CGT CAG CAT CAG CAA GAT GTC TG-3′). The expected amplicon size was approximately 770 bp, confirming the presence of PEDV RNA in the sample.
